# Effect of Imidazolium Nitrate Ionic Liquids on Conformational Changes of Poly(*N*-vinylcaprolactam)

**DOI:** 10.1021/acsomega.2c03650

**Published:** 2022-10-24

**Authors:** Reddicherla Umapathi, Krishan Kumar, Seyed Majid Ghoreishian, Gokana Mohana Rani, So Young Park, Yun Suk Huh, Pannuru Venkatesu

**Affiliations:** † NanoBio High-Tech Materials Research Center, Department of Biological Sciences and Bioengineering, Inha University, Incheon 22212, Republic of Korea; ‡ Department of Chemistry, University of Delhi, Delhi 110 007, India

## Abstract

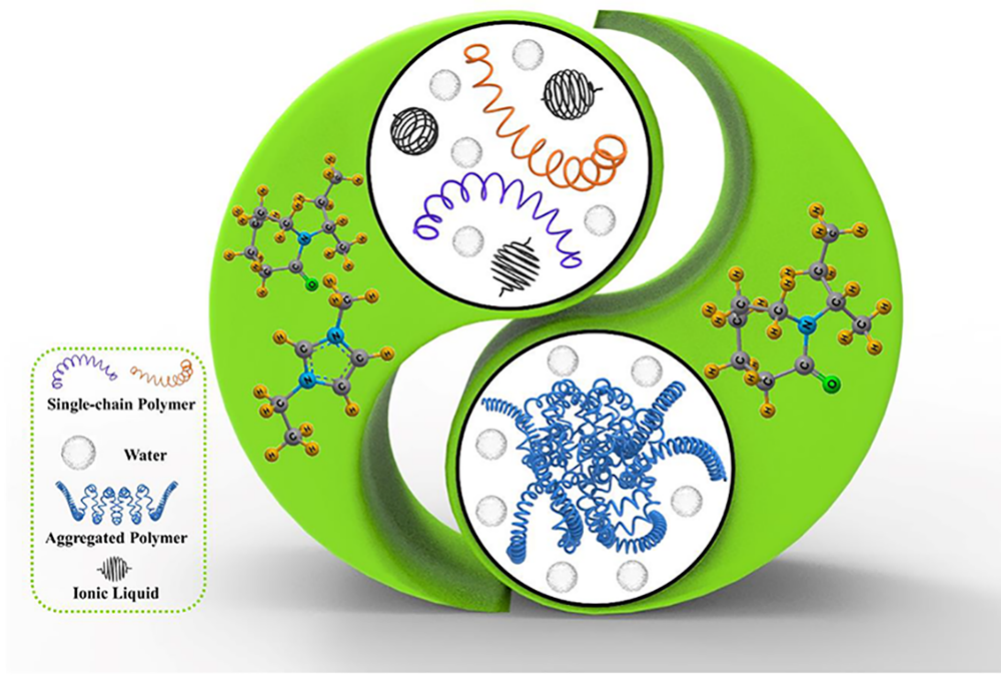

Detailed information about molecular interactions and conformational changes of polymeric components in the presence of ionic liquids (ILs) is essential for designing novel polymeric ionic liquid-based biomaterials. In biomaterials science and technology, thermoresponsive polymers (TRPs) are widely viewed as potential candidates for the fabrication of biorelated medical devices. Here, we synthesized thermoresponsive poly(*N*-vinyl-caprolactam) (PVCL) polymer and investigated the effects of imidazolium-based ILs (1-ethyl-3-methyl imidazolium nitrate and 1-butyl-3-methylimidazolium nitrate) with common anion and different cations on the phase transition behavior of PVCL aqueous solution. The impact of ILs on the phase transition behavior of PVCL was monitored by using UV–visible absorption spectra, steady-state fluorescence spectroscopy, thermal fluorescence spectroscopy, and temperature dependent dynamic light scattering. Results showed significant changes in the absorbance, molecular interactions, agglomeration, and coil to globule transition behaviors of PVCL in the presence of two ILs. PVCL aqueous solution showed significant conformational changes after the addition of ILs.

## Introduction

Stimulus responsive polymers (SRPs) or “smart” polymers are ubiquitous in nature and of particular interest in the biomedical and biotechnology fields. SRPs are intelligent systems that can respond promptly to the changes in surrounding environments by changing their properties, functionalities, conformations, solubilities, and structures. SRPs can be witnessed in everyday smart technologies. Understanding the nature of stimuli and the challenges associated with their synthesis can lead to the development of novel SRPs with enhanced stimuli in the nanoscale range and their adoption for real-world applications.^1−6^ Usually, SRPs are synthesized by free radical polymerization or using “advanced” living/controlled radical polymerization of monomeric units. Furthermore, they can be generated by incorporating “responsive” chemical functionalities into polymers. Stimulations of SRPs often manifest as changes in polymer conformations, which depend on chemistry of responsive polymer units.^7−9^

Among the different types of SRPs, thermoresponsive polymers (TRPs) have been widely studied for several decades due to their unique properties. TRPs display interesting properties, such as thermally triggered aggregation, contraction, and potential properties like gelation. Under all circumstances, temperature responsivity and sensitivity are well retained regardless of the geometric dimensions or topological structures of TRPs.^10−15^ These TRPs show major changes in conformation states when changes in surrounding environments are relatively modest. TRPs can be classified based on their solubilities. TRPs, like poly(*N*-isopropylacrylamide) (PNIPAM) and poly(*N*-vinylcaprolactam) (PVCL), have been used in various bioapplications, but understanding of the biophysical interactions between TRPs and ILs is limited.^16−18^

Ionic liquids (ILs) are ionic compounds comprising an organic cation and an inorganic or organic anion. Typically, ILs have weak Lewis basicities and acidities, and some melt at temperatures above 100 °C. Based on the principles of green chemistry, some ILs are regarded as excellent green solvents. The chief characteristic features of ILs include nonflammability, high electrochemical stability, high solubility in polymers, appreciable chemical stabilities, uniform interfacial ion arrangements, high thermal stabilities, and low toxicities, volatilities, flammabilities, and vapor pressures.^19−23^ Systematic synthesis using appropriate combinations of anions and cations has resulted in the syntheses of numerous ILs with different solvation powers, densities, conductivities, melting points, viscosities, polarities, acid and base characteristics, and hydrophobicities and hydrophilicities. Because of their polarities, some ILs are immiscible with most organic liquids, which makes them candidates for investigations on the phase transition and self-assembly behaviors of polymers and block copolymers. Due to their unique properties, a large number of organic electrolytes (commonly referred to as ILs) are now utilized in various scientific and technological fields. Specifically, imidazolium ILs have long alkyl side chains that can segregate to form polar and apolar domains.^23−26^

The unique molecular and biophysical interactions between TRPs and ILs constitute an emerging research area, and many investigations are now underway to advance the development of polymeric ionic liquids. When TRPs and ILs are mixed, they display different properties from those of pure solutions^26−28^ that are due to the formation of complex structures and specific aggregations. Lodge et al. investigated the self-assembly of amphiphilic diblock copolymers in ILs,^29−31^ and Early et al.^29^ studied the fragmentation kinetics of 1,2-polybutadiene-*block*-poly(ethylene oxide) micelles in imidazolium ILs. Kharel et al.^30^ investigated solution properties, such as the dynamic, structural, and thermodynamic properties, of poly(benzyl methacrylate) of different molecular weights in imidazolium and pyrrolidinium-based ILs using light scattering methods. Relevant dynamic and static properties were found to be functions of concentration, molecular weight, and temperature, and all systems exhibited LCST (lower critical solution temperature) behavior. In addition, these authors suggested that phase boundaries indicate a shift in critical composition toward poly(benzyl methacrylate) rich regions. Carrick et al.^31^ explained the LCST behavior of poly(benzyl methacrylate) in pyrrolidinium-based ILs. Turbidimetry analysis revealed that phase boundaries were strongly concentration-dependent. Recently Kumari et al.^32^ reported that ILs can efficiently increase the mobility of cells by reducing the elasticity of the lipid membrane, and that elasticity and mobility can be tuned by adjusting the IL concentration and cationic chain length. Yuan et al.^33^ deliberated on the polymerization, formation, mesostructuring, directional alignment, and self-assembly of poly(ionic liquid)s. Ueki et al.^34^ reported on the unique phase behavior of cross-linked polymer gels and linear polymers in ILs and observed that poly(benzyl methacrylate) and its copolymers demonstrated LCST-type phase separation in hydrophobic ILs. Furthermore, cross-linked poly(benzyl methacrylate) gels show discontinuous and reversible volume phase transition in imidazolium ILs on changing temperature. However, few studies have examined the effects of ILs on LCST of the PVCL. Here, we studied the effect of ILs on transition behavior of thermoresponsive PVCL using various biophysical methods.

## 2 Experimental Section

*N*-Vinylcaprolactam (VCL) (assay 98%), azobis(isobutyronitrile), and hexane (assay ≥97.0% (GC)) were acquired from TCI Chemicals (India) Pvt. Ltd. and recrystallized prior to use for polymerization. The fluorescent probe 8-anilino-1-naphthalenesulfonic acid (ANS; C_6_H_5_NHC_10_H_6_SO_3_H) (assay ≥97% (HPLC)) and the two ILs, that is, 1-ethyl-3-methylimidazolium nitrate ([Etmim][NO_3_]) (assay ≥99.0% (NT) and impurities ≤1.0% water) and 1-butyl-3-methylimidazolium nitrate ([Btmim][NO_3_]) (assay ≥95.0% (HPLC) and impurities ≤1.0% water), were acquired from Sigma-Aldrich and used as purchased. VCL was polymerized by free radical polymerization. The synthesis and characterization of PVCL was conducted as previously described.^35−37^ PVCL was synthesized by free radical polymerization of VCL using AIBN as initiator. After synthesis, polymer was precipitated with diethyl ether, filtered, and dried under vacuum. Successful synthesis of the PVCL was confirmed by ^1^H NMR and FTIR measurements; more detailed description about synthesis and characterization of PVCL can be obtained in prior articles.^2,35,36^ Appropriate amounts of PVCL and ILs were weighed with a Mettler Toledo analytical balance. Distilled water (Ultra 370 series, Rions India) was used to prepare the solutions. Spectroscopic studies were conducted at a PVCL concentration of 5 mg/mL and IL concentrations of 10, 15, or 20 mM. For UV–visible and fluorescence spectroscopy, ANS (the extrinsic probe) was added at low concentration (2 × 10^–5^ M) to avoid the probe influencing polymer aggregation. Double beam UV–visible spectrophotometer (UV-1800, Shimadzu Co., Japan), Cary Eclipse fluorescence spectrophotometer (Varian optical spectroscopy instruments, Mulgrave, Victoria, Australia) with an intense Xenon flash lamp as light source, and Zetasizer Nano ZS90 (Malvern Instruments Ltd., UK), equipped with He–Ne (4 mW, 632.8 nm) were used for UV–visible, fluorescence, and light scattering measurements, respectively. Furthermore, detailed specifications and instrumental details can be obtained in our previous articles.^37,38^

## 3 Results and Discussion

**Figure 1:**
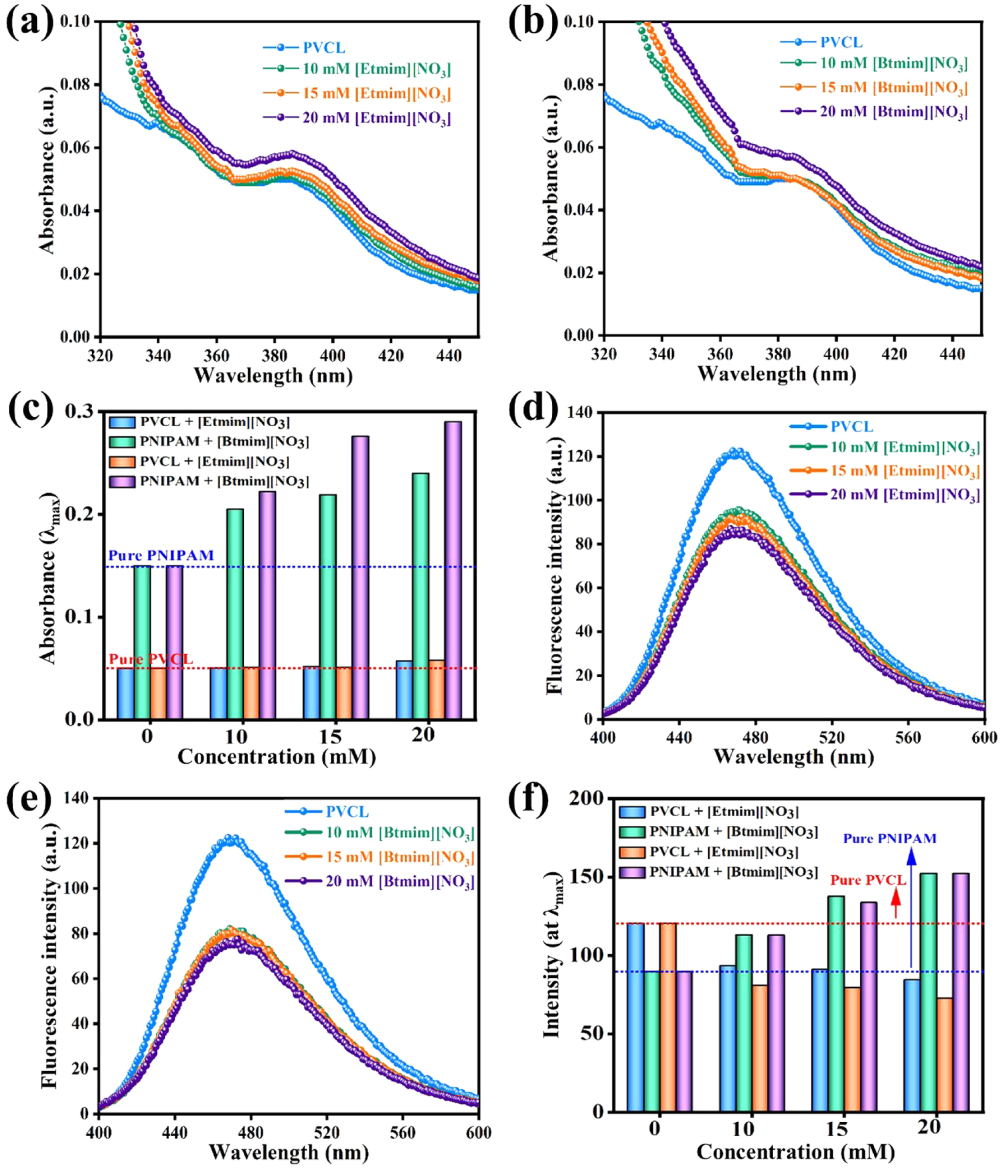
Changes in UV–visible absorption (a and b) and steady state fluorescence spectra (d and e) of ANS in aqueous PVCL solutions containing different concentrations of [Etmim][NO_3_] and [Btmim][NO_3_] at 25 °C. Comparative graph of absorbance (c) and fluorescence intensity (f) at maximum wavelength/intensity for PNIPAM and PVCL solution in the presence of different concentrations of imidazolium nitrate-based ILs.

In order to better understand the influence of imidazolium-based IL with varying alkyl chain length on the temperature-dependent transition of PVCL, we used absorption, steady state fluorescence, dynamic light scattering (DLS), and temperature-dependent fluorescence spectroscopy techniques. In addition, comparative analysis was performed between PVCL and PNIPAM to examine the effects of polymer structure on interactions between polymers and imidazolium nitrate-based ILs and provide mechanistic insight into the effects of ILs in mixed systems. UV–visible spectroscopy by sensitively responding to polymer structural changes provides useful indirect information. PVCL does not have a functional group that absorbs in the UV–visible region, and thus, we used ANS as an external probe to monitor IL-induced changes in PVCL. ANS mainly interacts with available hydrophobic sites and is useful for studying polymer thermal dehydration pathways. Figure 1(a and b) shows absorption spectra profiles of aqueous ANS-PVCL solution in the presence of various concentrations of imidazolium nitrate-based ILs. As shown in Figure 1(a and b, black line) in the absence of ILs, the ANS-PVCL solution had an absorption maximum (λ_max_) at ∼380 nm, which concurs with previously reported values.^35−37^ A slight absorbance increase was observed after adding [Etmim][NO_3_] to ANS-PVCL solution at relatively low concentrations (10 or 15 mM). However, a significant increase in absorbance with no shift in λ_max_ was observed in the presence of 20 mM. On the other hand, as demonstrated in Figure 1(b), addition of [Btmim][NO_3_] at 10 or 15 mM had no significant effect on absorbance or λ_max_, whereas at 20 mM it increased absorbance but did not affect wavelength. These changes in absorption spectra depend on alterations in the ANS microenvironment. Minimum absorbance was obtained for ANS-PVCL solution, and slight increases were observed at higher IL concentrations, which suggested structural variations in PVCL. Figure 1(c) shows UV–visible spectroscopy absorbances obtained at wavelength maximum for PNIPAM and PVCL in the aqueous solution in the presence of different concentrations of imidazolium nitrate-based ILs.

Steady state fluorescence spectroscopy was employed to scrutinize the impact of ILs on the conformational transition of PVCL because it provides information related to the solvation behavior of IL-PVCL mixtures. Due to the absence of a fluorescent component in PVCL, ANS was used as a probe. Figure 1(d and e) shows the emission spectra profiles of ANS-PVCL aqueous solutions in the presence or absence of imidazolium nitrate-based ILs. In the absence of an IL, ANS-PVCL solution had an emission intensity maximum at ∼475 nm, which was consistent with the existing literature.^35^ Emission intensity maximum of ANS-PVCL aqueous solution depends on the chain length and molecular weight of the polymer. Fluorescence intensity decreased from 121 to 95, 89, and 86 a.u. after adding 10, 15, or 20 mM of [Etmim][NO_3_]. On the other hand, PVCL emission intensity decreased from 121 to 81, 80, and 75 a.u. after adding 10, 15, or 20 mM of [Btmim][NO_3_], respectively. ANS-PVCL solution had the highest fluorescence intensities across the entire wavelength range, which showed that polymer solutions were more hydrophobic in the absence of ILs. Fluorescence intensity quenching signifies a change in polarity around the probe’s microenvironment. Furthermore, a greater decrease in intensity was observed for [Btmim][NO_3_] than [Etmim][NO_3_] IL. Figure 1(f) shows the maximum fluorescence intensities for PNIPAM and PVCL in aqueous solution in the presence of different concentrations of imidazolium nitrate-based ILs.

**Figure 2:**
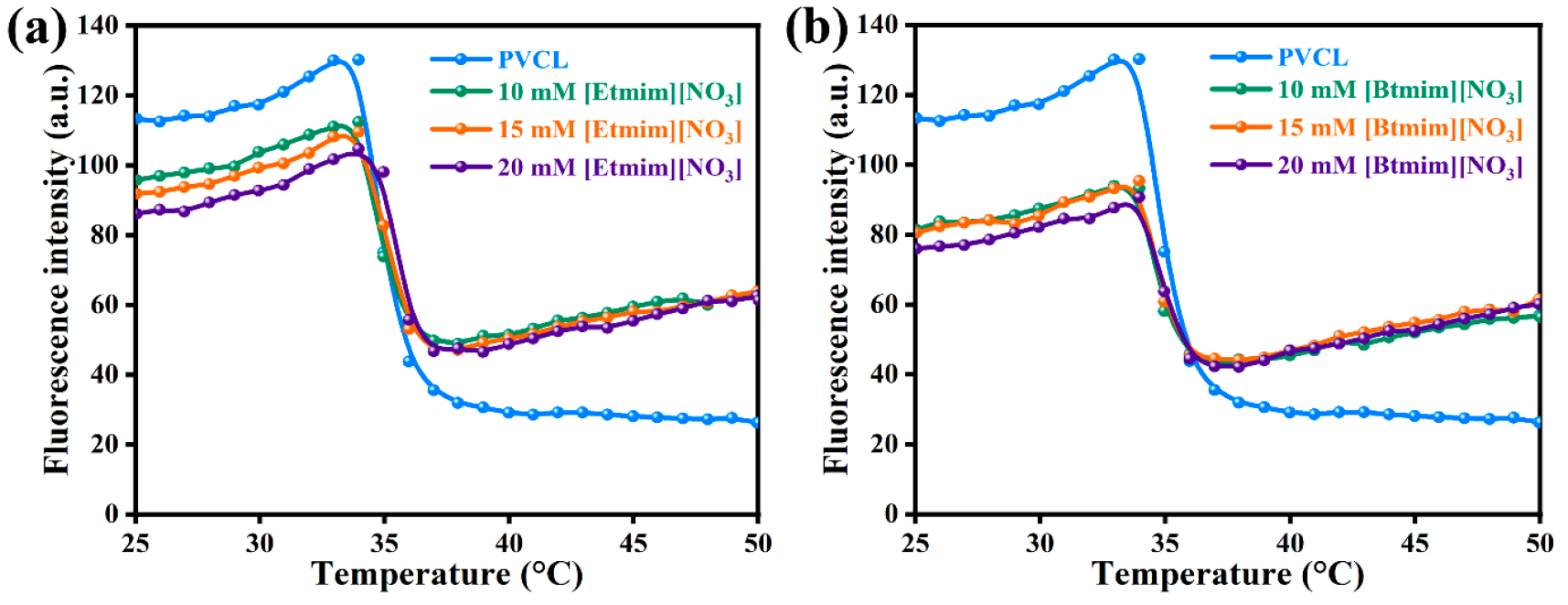
Temperature dependent fluorescence spectroscopy of PVCL aqueous solutions containing different concentrations of (a) [Etmim][NO_3_] or (b) [Btmim][NO_3_].

**Scheme 1:**
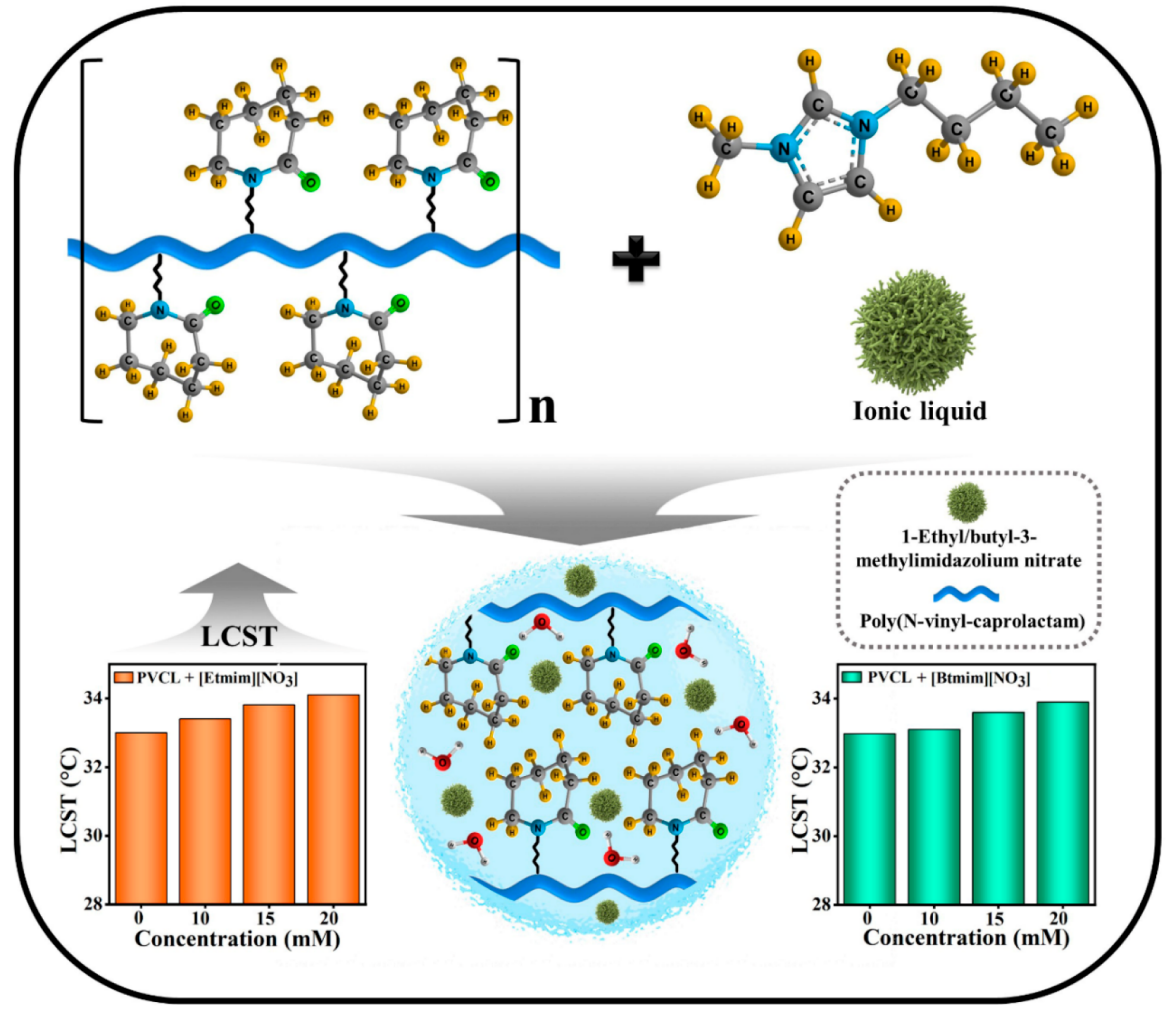
Schematic Illustration Depicting the Changes and Interactions Occurring in PVCL Solution with Addition of [Etmim][NO_3_] or [Btmim][NO_3_] ILs LCST values were determined by using temperature-dependent fluorescence spectroscopy.

The thermal fluorescence spectroscopy technique was used to ascertain the influence of ILs on thermally induced structural variations in PVCL solution. Phase transition behavior or LCST of PVCL was systematically examined by measuring initial breakpoints (sudden changes) in the fluorescence intensity on changing temperature. Figure 2 shows the temperature dependent fluorescence intensity of PVCL from 25 to 48 °C in the presence or absence of imidazolium nitrate-based ILs. For PVCL solution, a sudden decrease in fluorescence intensity was observed at ∼33.5 °C (LCST), which agrees with previous reports^35−37^ which may have been due to disruption of light passage due to turbidity formation. The presence of [Etmim][NO_3_] at 10, 15, or 20 mM increased the LCST to 33.7, 33.9, and 34.6 °C, respectively. These variations in LCST were possibly due to the formation of hydrogen bonds between IL ions and water in the structures present around PVCL. The presence of IL indirectly influences PVCL–water interactions and ultimately affects LCSTs. On the other hand, in the presence of [Btmim][NO_3_] at 10, 15, or 20 mM only small increases in transition temperature were observed, and LCST values increased from 33.5 to 33.6, 33.7, and 33.8 °C, respectively. The smaller increases in LCST values observed in the presence of [Btmim][NO_3_] may have been due to the presence of a larger alkyl chain in the IL cationic group. LCST values obtained by temperature-dependent fluorescence spectroscopy are summarized in Table 1. Similar increases in transition temperature were observed by Venkatesu et al. for aqueous PVCL solution in the presence of imidazolium chloride-based ILs.^36^ Recently, Umapathi et al.^39^ also reported an increase in transition temperature for aqueous PNIPAM solutions in the presence of similar imidazolium nitrate-based ILs. Moreover, a similar trend of transition temperature variations was observed for ILs with various alkyl chain lengths. Li et al.^40^ also studied the phase transition behavior of poly(ethylene oxide) in the presence of imidazolium tetrafluoroborate and noticed a decrease in transition temperature on increasing PEO content. Scheme1 schematically represents the changes and interactions occurring in PVCL solution with the adding of [Etmim][NO_3_] or [Btmim][NO_3_] ILs. Graphical bar diagrams of LCST values were determined by using temperature-dependent fluorescence spectroscopy.

**Table 1:** Phase Transition Temperatures of PVCL in the Presence of Imidazolium Nitrate-Based ILs, as Determined by Thermal Fluorescence Spectroscopy

	LCST (°C)	
Concentration of IL (mM)	[Etmim][NO_3_]	[Btmim][NO_3_]
0	33.5 ± 0.1	33.5 ± 0.1
10	33.7 ± 0.3	33.6 ± 0.3
15	33.9 ± 0.1	33.7 ± 0.1
20	34.6 ± 0.3	33.8 ± 0.2

**Figure 3:**
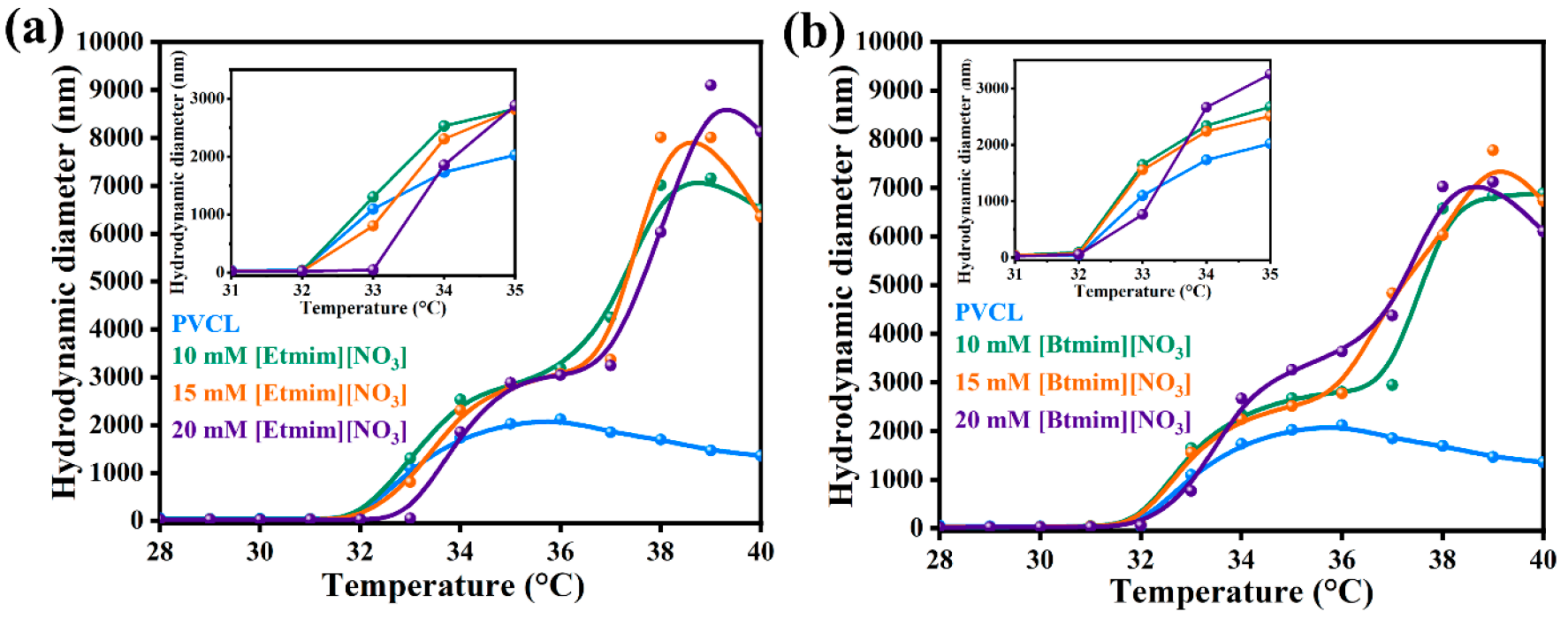
Temperature dependent hydrodynamic diameter of PVCL aqueous solution containing different concentrations of (a) [Etmim][NO_3_] and (b) [Btmim][NO_3_].

DLS analysis when performed at various temperatures can provide more information related to the size and aggregation phenomenon of PVCL in the presence of imidazolium nitrate based ILs. Figure 3 shows variations of PVCL hydrodynamic diameters (*d*_H_) in aqueous solutions in the presence of ILs. This variation in size (*d*_H_) is important when considering the effects of IL additions on LCST values. After the temperature-dependent hydrophobic collapse of PVCL, particle size suddenly increased due to agglomerate formation. In the absence of IL (PVCL solution), this abrupt increase was observed at ∼32.0 °C, which is consistent with the previous results.^36^ However, after adding [Etmim][NO_3_] at 15 and 20 mM, the transition temperature increased from 32.0 to 32.3 and 33.0 °C, respectively. On the other hand, after adding [Btmim][NO_3_] at 20 mM, LCST increased from 32.0 to 32.3 °C. These results demonstrate that PVCL hydration occurs at a slightly higher temperature in the presence of higher amounts of ILs. This increase in transition temperature can be ascribed to an interaction between ILs and hydration layer around polymer and subsequent disruption of intermolecular interactions with PVCL segments. This rearrangement in the hydrogen bonding interactions triggers delayed aggregation of the polymer aggregation, and the phenomenon is more prominent in the presence of higher IL concentrations.

One can conclude that imidazolium nitrate-based ILs act to stabilize the solvated state of PVCL in water. Furthermore, variations in agglomerate formation were only observed in the presence of an IL; ILs themselves did not aggregate in solution on increasing temperature. Trends shown by DLS-obtained LCST values of PVCL-IL mixtures agreed well with temperature-dependent fluorescence spectroscopy results. Wang et al.^41^ deliberated on the gelation microdynamics of PNIPAM in the presence of 1-ethyl-3-methylimidazolium bis(trifluoromethyl sulfonyl)imide ILs, and the role played by 1-butyl-3-methylimidazolium tetrafluoroborate on LCST transition of the PNIPAM, and described the concentration-dependent impact of ILs on phase behavior of PNIPAM.^42^ Moreover, Kohno et al.^43^ studied the temperature-sensitive phase transition of poly(ionic liquid)-aqueous mixed systems, and Liu et al.^44^ studied the LCST behavior of ionogels consisting of polyacrylates and hydrophobic 1-alkyl-3-methylimidazolium bis{(trifluoromethyl) sulfonyl} amide ILs and demonstrated the tuning effect of mixing ratio on LCSTs. These studies demonstrated the effect of ILs on the thermal transitions of polymer/ionic liquid mixed systems. Table 2 shows transition temperature values of PVCL solutions derived by DLS.

**Table 2:** Phase Transition Temperatures of PVCL in the Presence of the Two ILs as Determined by Dynamic Light Scattering Studies

	LCST (°C)	
Concentration of IL (mM)	[Etmim][NO_3_])	[Btmim][NO_3_]
0	32.0	32.0
10	32.0	32.0
15	32.3	32.0
20	33.0	32.3

**Scheme 2:**
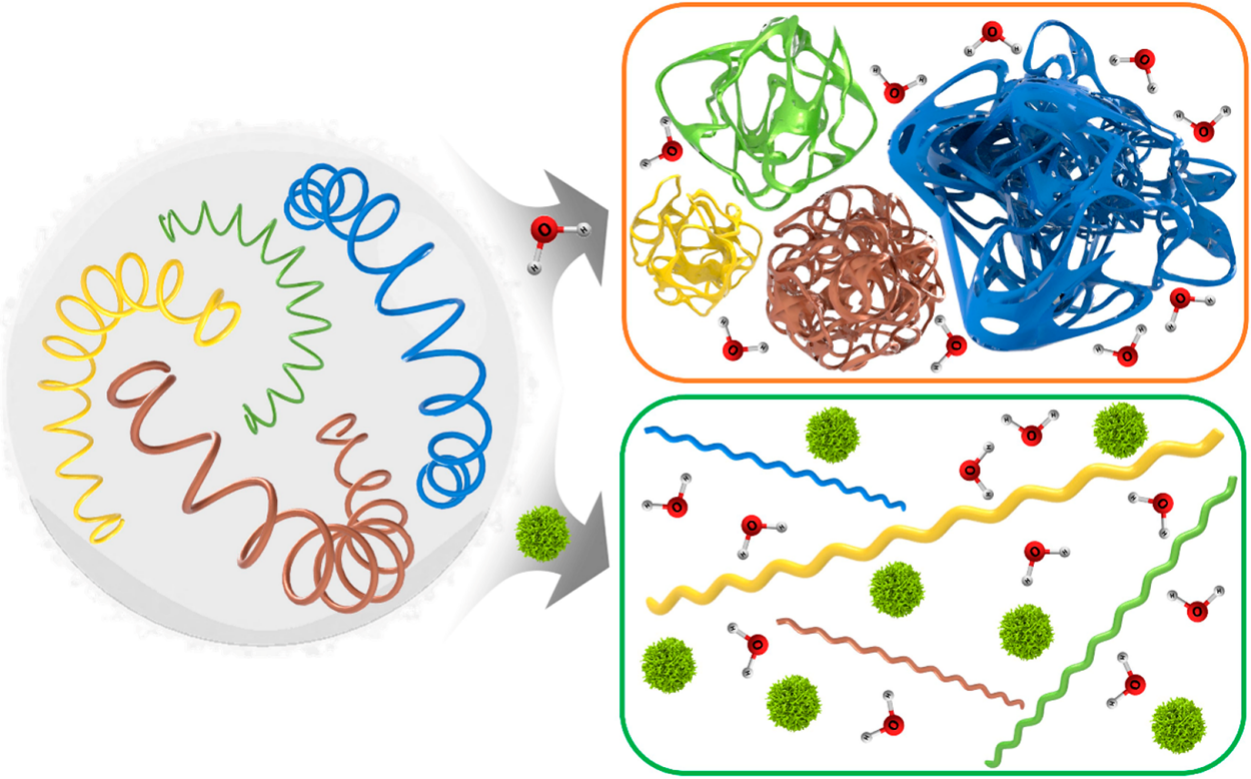
Schematic Representation of the Conformational Changes Occurring in Aqueous [Etmim][NO_3_] or [Btmim][NO_3_]/PVCL Systems

Our results show that the addition of ILs increases the transition temperature of PVCL aqueous solution, which suggests ILs behave as a constructor for the hydration layer of PVCL. Scheme 2 schematically represents the conformational changes believed to occur in aqueous [Etmim][NO_3_] or [Btmim][NO_3_]/PVCL systems.

**Figure 4:**
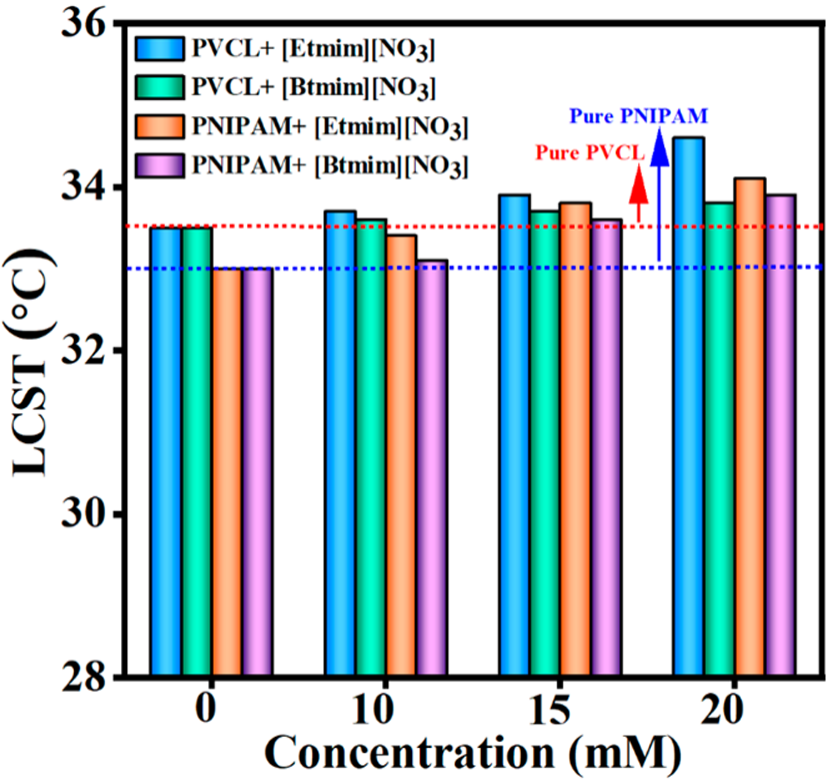
Transition temperatures of PVCL (present work) and PNIPAM in aqueous solution^39^ in the presence of various concentrations of [Etmim][NO_3_] or [Btmim][NO_3_] as determined by temperature-dependent fluorescence spectroscopy.

A comparison of the obtained results with those of aqueous solutions of PNIPAM was performed to better understand the influence of imidazolium based ILs (Figure 4). Similar variations in transition temperature were observed in the presence of 20 mM of [Etmim][NO_3_] or [Btmim][NO_3_] (LCST increased from 33.0 to 33.9 and 34.2 °C, respectively).^39^ On the other hand, a decrease in transition temperature was noticed for PNIPAM in the presence of [Etmim][Cl] or [Btmim][Cl], respectively,^36^ whereas the transition temperature of PVCL increased in the presence of [Etmim][NO_3_] or [Btmim][NO_3_].^36^ This comparison illustrates that the studied ILs in the presence of PVCL is behaving as the “constructors” for hydration layer of PVCL, whereas the same ILs disrupt the hydration layer of PNIPAM in aqueous solution.

## 4 Conclusions

The present research shows the effects of varying the length of the alkyl chain of the cationic group of 1-alkyl-3-methyl imidazolium nitrates on the conformational transition behavior of PVCL. Different spectroscopic techniques such as UV–visible, steady-state fluorescence, temperature-dependent fluorescence spectroscopy, and DLS were used to study the transition temperature of the PVCL polymer in aqueous solution in the presence of ILs. The results of spectroscopic techniques revealed that imidazolium nitrate-based ILs protect the hydrated structure of PVCL. Moreover, UV–visible and steady state fluorescence spectroscopy results showed that IL variations altered hydrogen-bonding interactions and thus the solvation behavior of PVCL. Thermal DLS and fluorescence spectroscopy results showed LCST values of the PVCL increased in the presence of ILs. Furthermore, a comparison with PNIPAM/IL mixtures showed that polymer structure influenced interactions with imidazolium ILs containing ethyl and butyl groups. Our results suggest that variations of alkyl chain length of cation alter the transition behavior of the PVCL due to variations in the hydrogen bonding network around PVCL particles in aqueous solution. We hope our findings will aid the development of novel biocompatible materials and smart drug carriers that can be induced to aggregate by simply adding ILs as cosolvents.
